# Formulation and characterization of exenatide-loaded PLGA microspheres prepared by coacervation

**DOI:** 10.1007/s13346-025-02008-2

**Published:** 2025-12-09

**Authors:** Cameron White, Steven P. Schwendeman

**Affiliations:** 1 Department of Pharmaceutical Sciences, Biointerfaces Institute, University of Michigan, Ann Arbor, MI 48109 USA; 2 Department of Biomedical Engineering, University of Michigan, Ann Arbor, MI 48109 USA

**Keywords:** Bydureon, PLGA, Coacervation, Phase separation, GLP-1, Controlled release, Initial burst

## Abstract

Bydureon^®^ is a once-weekly injection of poly(lactide-*co*-glycolide) (PLGA) microspheres containing exenatide acetate, a synthetic analog of the GLP-1 receptor agonist exendin-4. These microspheres are formulated by coacervation (i.e., phase separation), using a single-emulsion method. There remains a knowledge gap between how formulation variables affect product attributes and performance. We aimed to bridge this gap by evaluating the effect of formulation variables on encapsulating exenatide in PLGA microspheres at similar compositions to Bydureon^®^. We first screened process variables without peptide to establish stability windows during coacervation, i.e., conditions that produced high yields of well-formed microspheres. We introduced exenatide during coacervation as a function of PLGA concentration, DCM (dichloromethane): water and DCM: Si oil (polydimethylsiloxane) volume ratios, hold time between Si oil addition and heptane bath immersion, and other manufacturing conditions. We evaluated the formulation yield, residual solvent content, encapsulation efficiency, and 24-h release. A PLGA concentration of 6% w/w was selected because of its wide range of stable formulations with varying DCM: Si oil phase volume ratios. The hold time between Si oil addition and heptane immersion was set at 1 min, although microspheres were stable between a range of 10 s to 2 min. The resultant formulations displayed elevated yields of > 50%, and a low in-vitro 24-h burst release of 2–6%. These formulations exhibited continuous release profiles of predominantly parent and glycolic acid acylated peptide for over 56 days in vitro, as expected by the commercial product. The framework of conditions and their effects on the formulations was established for loading exenatide in PLGA microspheres with desirable release characteristics. These results are useful for both microencapsulation of generic and new peptides in PLGA microspheres by coacervation.

**Graphical abstract:**

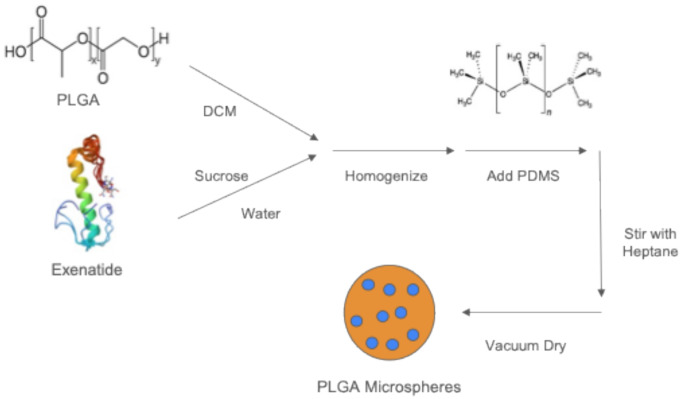

**Supplementary Information:**

The online version contains supplementary material available at 10.1007/s13346-025-02008-2.

## Introduction

 Long-acting release systems (LAR) based on poly (lactic-co-glycolic acids) (PLGAs) are an important class of dosage forms that aim to improve the safety and efficacy of the medications by decreasing the frequency of administrations, which improves patient compliance and outcomes. PLGA microsphere products such as Sandostatin^®^ and Lupron DepotⓇ are LAR systems that encapsulate octreotide and leuprolide, respectively. These FDA-approved products have been commercial successes for the last 25–35 years. While octreotide and leuprolide are comparably small peptides with fewer than 10 amino acid residues, PLGA can be used to encapsulate much larger polypeptides, such as glucagon-like-peptide 1 receptor agonists (e.g., exenatide acetate) and proteins [1].

Exenatide is a synthetic analog of a naturally occurring peptide found in the saliva of the Heloderma lizard. This peptide is a GLP-1 receptor agonist and is responsible for increasing glucose-dependent insulin secretion from the pancreas, suppressing glucagon secretion from the pancreas, as well as delaying gastric emptying [2]. Subcutaneous injections of exenatide, marketed as Byetta^®^, has a short half-life of 2 h, and therefore requires a strict dosing interval of 1 h before meals twice daily [3]. Bydureon^Ⓡ^ is a once-weekly subcutaneous injection of exenatide encapsulated in PLGA microspheres. The main components of Bydureon^Ⓡ^ microspheres are carboxylic acid-terminated 50:50 PLGA, exenatide acetate, and sucrose [3]. Bydureon^Ⓡ^ is intended to provide an alternative to patients who are uncomfortable with the frequency of injections or suffer from frequent inflammation at the injection sites. Encapsulating exenatide within a polymer matrix reduces the severity of injection site inflammation and nausea, which are commonly experienced by patients that are prescribed Byetta^Ⓡ^ [2]. Bydureon^Ⓡ^ was first approved by the FDA in 2012 with an aqueous injection vehicle [4] and thereafter updated by improved packaging in a pen. This second product was discontinued by the manufacturer and the product is now used in the form of Bydureon BCise^Ⓡ^, which include the same microspheres as Bydureon^Ⓡ^, but stored and administered in an oily triglyceride vehicle, also within a pen injector. Bydureon BCise^Ⓡ^ has the advantage relative to the original Bydureon^Ⓡ^ of not requiring titration or resuspension of particles by the patient.

While PLGA microspheres and its properties during formulation and release has been extensively studied, most literature focuses on formulations prepared by the common double-emulsion water-in-oil-in-water (W/O/W)-solvent evaporation methods [5]. Bydureon^®^, on the other hand, is formulated by the lesser-reported process of coacervation. Coacervation is a complex physical process that involves the slow desolvation of PLGA and the subsequent accumulation around the emulsified exenatide-containing water phase. This desolvation is initiated and sustained by the addition of a nonsolvent—typically polydimethylsiloxane (PDMS), which is a third solvent that reduces the solubility of PLGA in dichloromethane (DCM). PLGA is insoluble in PDMS, while PDMS and DCM are fully miscible [6].

As more PDMS is added to the system, the PLGA slowly precipitates out of solution onto the dispersed water droplets containing the water-soluble peptide drug. Microspheres prepared by coacervation possess both many similar, but also significantly different, attributes compared to solvent evaporation methods of formulation. Chiefly among the different attributes for coacervation-produced microspheres is the use of a single water-in-oil emulsion (W/O). Similarly, the particles are formed in oil instead of water as for solvent evaporation. These differences typically result in higher encapsulation efficiencies and lower burst release [7]. Additionally, microspheres formulated via coacervation require precise conditions of the phase separation, which are more difficult to control than those process conditions used during solvent evaporation. In addition, the microspheres typically retain multiple additional residual solvents, e.g., heptane and silicone oil as well as the carrier solvent, dichloromethane. These additional factors present extra challenges during formulation development and scaling up to large scale manufacture.

There is currently a knowledge gap in the scientific literature between the coacervation-specific formulation parameters and how they affect key product attributes. The purpose of this paper was to help bridge this knowledge gap. To investigate this microencapsulation method, the coacervation process was performed on a bench scale. Key formulation parameters were identified and optimized to maximize yield and minimize burst release. Minimizing residual solvents such as DCM is also critical since many organic solvents are unsafe when introduced to the body beyond specified levels. Some residual solvents are also capable of plasticizing the polymer, which can contribute to increased water ingress and elevated burst release [8, 9]. Exenatide is FDA-indicated to improve glycemic control in adult patients with type 2 diabetes mellitus when used as an adjunct to diet and exercise. It is not recommended as first-line therapy to treat diabetes. However, clinical trials have shown exenatide to be both safe and effective when used as monotherapy or in combination with other diabetic medications [10]. Minimizing burst release of exenatide is important to reduce side effects such as nausea, and inflammation at the injection site. Maximizing yield and encapsulation efficiency is important to conserve expensive drug. Finally, a proper investigation into the chemical stability of exenatide within PLGA microspheres formulated via coacervation is of importance to ensure the safety and efficacy of the final product.

## Materials and methods

### Chemicals

Exenatide acetate was supplied by Bachem Americas (Torrance, CA, USA). Poly(D, L-lactide-co-glycolide) 50:50 polymer (acid-terminated, MW ~ 24k-38k) RG 503 H was purchased from Evonik Industries (Essen, Germany). Dichloromethane was provided by Merck Corporation (Rahway, NJ, USA). Polydimethylsiloxane of varying viscosities were supplied by Sigma Aldrich (St. Louis, MO, USA). Sucrose, ultrapure water, and heptane were HPLC grade, and were supplied by Fisher Scientific (Waltham, MA, USA). Filter paper (nylon, 0.45 μm) was supplied by Micron Separations (Westboro, MA, USA).

### Formulation of blank microspheres by coacervation

Blank microspheres without peptide were prepared initially to determine conditions for successful microsphere formation. Before coacervation, a water-in-oil (W/O) emulsion was prepared (no peptide) as would be needed to suspend exenatide throughout PLGA oil phase for peptide-loaded microspheres. The water phase consisted of 660 µL of double distilled water. The oil phase of the primary emulsion consisted of 462–975 mg of PLGA, dissolved in 6.6 mL of DCM. These two phases were combined (W in O) and emulsified in 23-mL culture tubes for 20 min at 14,000 rpm by a Polytron PT 2500e homogenizer, supplied by Kinematica (Lucerne, Switzerland). During homogenization, the mixture was placed into an ice bath to help dissipate the heat liberated by the homogenizer. The resultant emulsion was placed in a 100 mL secondary beaker suspended in an ice bath and was stirred at 600–900 rpm for 1 min by an IKA Eurostar 20 overhead stirrer supplied by the Ika Company equipped with a 5 cm rotor impeller (Staufen, Germany). 1–15 mL of PDMS was added dropwise with a dispensing speed of 0.5-4 mL/min into the stirring emulsion with a Fisherbrand single syringe pump (Fisher Scientific). After the conclusion of PDMS addition, the coacervate was held for 0.5–2 min before being slowly decanted into a 500 mL heptane bath, that was held at 4–20 °C by a ThermoFisher RTE-111 refrigerated circulator. The embryonic microspheres were hardened by the heptane bath stirred at 550–1150 rpm for 30 min. The microspheres were sieved from 20 to 125 μm and vacuum dried for 1–2 days. Yield was determined by gravimetry from the sieved fraction. The specific values of formulation parameters were determined by making particles at a constant composition while altering one parameter at a time. Observations of polymer aggregation combined with yield values were used to create rough stability windows for encapsulation (*see below*).

**Fig. 1:**
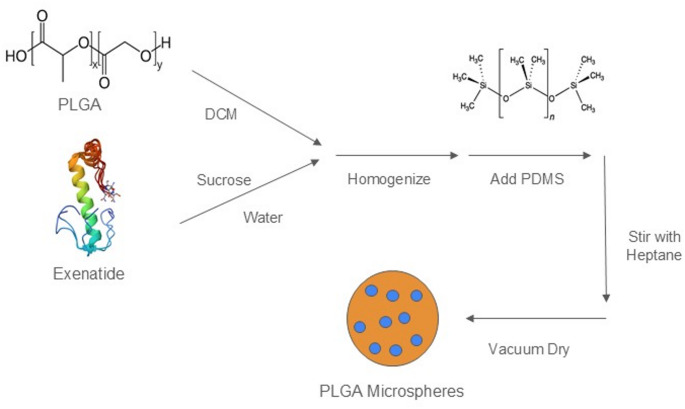
Formulation schematic displaying the procedural steps in generating microspheres by coacervation

### Formulation of exenatide-containing microspheres by coacervation

PLGA microspheres containing exenatide were prepared as above for blank microspheres (Fig. 1) with the following differences. The water-phase of the primary emulsion consisted of 330–720 µL of water, 32 mg of exenatide acetate, and 12 mg of sucrose.

The microspheres were formulated by a single emulsion water-in-oil (W/O) preparation method. The oil phase of the primary emulsion consisted of 560 mg of PLGA, dissolved in 6.6 mL of DCM. The water phase consisted of 330–720 µL of water, 32 mg of exenatide acetate, and 12 mg of sucrose. The phases were emulsified for 10–20 min at 10,000–20,000 rpm in 23-mL culture tubes. All formulation parameters not mentioned were identical to the blank microsphere formulation procedure.

### Determination of drug loading

Loading of exenatide into the microspheres was determined by Ultra performance liquid chromatography (UPLC). A single-phase extraction method was used to prepare microsphere samples for analysis. 5 mg of microspheres were dissolved in acetonitrile, and vortexed for 10 min. A second phase of 1.2 mL of 50 mM sodium acetate buffer pH 4 was added and was vortexed for another 10 min. The mixture was centrifuged at 10,000 rpm for 20 min to sediment the now insoluble PLGA. The supernatant was sampled and tested by UPLC (Waters Acquity H-Class UPLC Plus). The mobile phase consisted of v/v 0.1% formic acid (FA) in acetonitrile and 0.1% FA in water and the column was a Waters Corp ACQUITY UPLC Protein BEH C4 Column, 300Å, 1.7 μm, 2.1 mm X 50 mm. Flow rate was 0.4 ml/min, injection volume was 10 µL and detection wavelength was set to 280 nm. A linear gradient of 25–35% ACN with 0.1% (v/v) formic acid over 2 min at a flow rate of 0.5 mL/min was used.

### Characterization of morphology, and particle/pore size

Morphology, size distribution, and pore size distribution were characterized by scanning electron microscopy (SEM). Dry microsphere samples were adhered to aluminum stubs using double-sided tape. The microspheres were sputter coated with gold, and analyzed with a TESCAN Mira3 FEG SEM and images were recorded at 135x magnification and 5 kV. Surface pore size was estimated by the imaging software’s length acquisition tool.

### Determination of residual solvent by GC

Residual solvent levels of DCM and heptane were quantified by headspace gas chromatography (GC) (ThermoScientific Trace 1310 Gas Chromatograph). Samples were prepared by dissolving 10 mg of dry microspheres in 1 mL of dimethylsulfoxide (DMSO). The samples were applied to the GC by headspace injection, the GC conditions were as follows: nitrogen gas as the carrier solvent at a flow rate of 25 mL/min with air and hydrogen flow rates of 350 mL/min and 35 mL/min, respectively; the front detector temperature was 240 °C and the front inlet pressure was a constant flow at 2 mL/min. Each sample was agitated for 20 min at 80 °C and 1 mL of the headspace sample was injected into the front inlet with the temperature at 140 °C with a split flow rate of 40.0 ml/min, and a split ratio of 20. The GC column temperature was initially set at 40 °C for 15 min, then increased at 10 °C/min to 240 °C and held at 240 °C for 2 min. A standard curve was prepared by adding the desired solvent to DMSO at 1, 10, 25, 50, and 100 ppm.

### Determination of primary emulsion size by DLS

Primary emulsion size was determined by dynamic light scattering (DLS) using a Malvern Zetasizer Nano ZS. The detector was placed at a 173º backscatter. The primary emulsion was diluted with dichloromethane at a 1:10 ratio, and then was added to a ZEN0040 cuvette. The sample was held at 4 °C. The emulsion size was averaged over 10 runs.

### Determination of initial and long-term release kinetics, water uptake, and mass loss

Release of exenatide from the microspheres was determined indirectly by the remaining peptide in the polymer by two separate methods. For the initial burst release, 5 mg of microspheres were placed into 1.5 mL Eppendorf tubes. The microspheres were suspended in 1 mL of phosphate-buffered saline (PBS) with 0.02% v/v Tween 80 and 0.02% w/w sodium azide at pH 7.4. The particles were then incubated for 1 day at 37 °C under gentle agitation. The particles were then centrifuged at 13,500 g, and the supernatant was aspirated. The tubes were then dried in a vacuum oven at room temperature for 2 days. The remaining exenatide content in the particles were then analyzed by the above UPLC method and compared to the initial loading values. For long-term release studies, timepoints were generated for 1, 3, 7, 14, 21, 28, 35, 42, 49, and 56 days in triplicate. 10 mg of microspheres were suspended in 1 μm pore nylon filter bags provided by The Cary Company (IL, USA). The bag itself was suspended in the release vessel with PTFE tape to ensure total freedom of fluid diffusion. The bags were immersed in 25 mL of identical release media and were incubated at 37 °C with gentle agitation. At each time point, the corresponding bags were removed from the incubator, and the particles were collected. The bags were dismantled to ensure that all the particles were collected. The particles were pipetted onto the wet filter, and then dried in a vacuum oven at room temperature for 2 days. Exenatide was extracted from the dried particles by a single-phase extraction with acetonitrile and 50 mM sodium acetate pH 4.5 buffer at a 1:4 v/v ratio. The supernatant was collected and analyzed by UPLC at 280 nm.

## Results and discussion

### Initial formulation screening and identification of a stability window

The stability window of coacervation, a set of conditions during formulation that prevent irreversible aggregation of the polymer, is a critical parameter of formulation development that governs many key attributes of the final microspheres. Typically, the stability window is displayed as a ternary phase diagram, with PLGA, PDMS, and DCM concentrations as the three axes. Merkle et al.. describes the rheological steps of coacervation as follows: (1) pseudoemulsion of PDMS droplets within the polymer-rich phase; (2) true mixing of the three phases into a ternary system where there are PDMS droplets within the polymer rich phase as well as DCM droplets within PDMS; (3) phase inversion and separation, leading to the formation of unstable microdroplets that start to flocculate; (4) formation of a stable dispersion of coacervate droplets; and (5) irreversible aggregation and precipitation of polymer droplets [11]. Ideal formulations, which are those that fall within the stability window, do not experience the fifth rheological step, which inevitably leads to decreased yields and formulation viability.

**Fig. 2:**
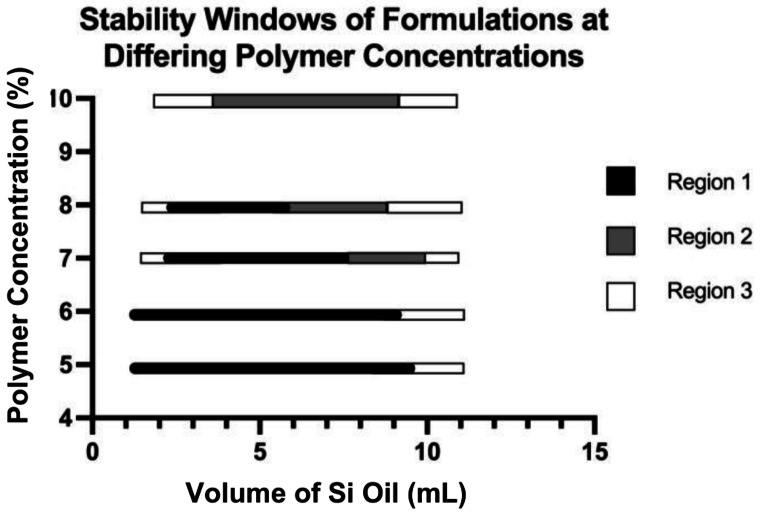
Stability windows of formulations prepared with varying polymer concentrations. *Region 1*: This region represents high yield (> 50%) and minimal aggregation or precipitation. *Region 2*: This region represents high yield (> 50%), minimal aggregation or precipitation, but pronounced flocculation after drying. *Region 3*: This region represents lowered yield (25–50%) due to increased aggregation or precipitation. Outside of the bars represents volumes of Si oil that failed to produce meaningful quantities of microspheres

As shown in Fig. 2 the PDMS: DCM ratio was of vital importance for achieving the stability window. As polymer concentration was increased just a few percent (from 5 to 10%) the stability window shrank significantly from the desirable *Region 1* (high yield, minimal aggregation or precipitation, minimal flocculation on drying) for 1–9 mL PDMS to undesired *Regions 2* (high yield, minimal aggregation or precipitation, significant flocculation on drying) and *3* (Low yield, high aggregation or precipitation). PLGA concentration had no effect on microsphere size, as shown in Fig. 6. Note that the formulation with a 9% PLGA concentration was not evaluated as the 10% formulation adequately showed the trend.

As shown in Figure S1a, a light microscope image of the coacervate created under conditions outside the stability window. There were large contiguous phases of PLGA/dichloromethane and Si oil, and desolvation had not occurred. There were also small pockets of isolated microspheres, but the majority of polymer was still not dispersed. The threshold between insufficient and sufficient Si oil volume was observed to be abrupt. For example, formulations prepared with 1.25 mL of Si oil completely aggregated while immersed in the heptane bath, whereas formulations prepared with 1.5 mL of Si oil dispersed properly. Interestingly, at higher polymer concentrations (≤ 7%), there was a small Region 3 at low and high PDMS addition levels before a proper stability window was reached. Microspheres in this region initially aggregated during the heptane wash, but gradually separated into dispersible particles by the end of the wash. Consequently, these particles exhibited deformed morphologies and significantly larger volume mean diameters. Figure S1b represents the newly formed coacervate within the stability window. While there were still continuous regions of the Si oil, there were numerous embryonic microspheres of varying sizes. As polymer concentration was increased, *Region 1* narrowed, and vanished at a concentration of 10% w/v. Higher polymer concentration is expected to result in more frequent microsphere collisions and adherence, thus leading to higher aggregation and lower yield. *Region 2* was a difficult region to accurately assess. Formulations in this region behaved similarly to those in *Region 1* for the entire process except for the final vacuum drying step. Due to less advanced drying methods (static drying in a vacuum oven), it is possible that *Region 2* could produce stable microspheres if proper commercial drying equipment were used. According to the patent literature from the Bydureon innovators, it is suggested that the particles were dried with a SWECO PharmASep™ dryer, which utilizes vibrating screens to minimize microsphere aggregation and lumping.

Figure S1c represents the newly formed coacervate outside the stability window. The polymer in this case aggregated to such an extent that the polymer coalesced into a single mass, rather than a suspension of particles.

Once a stability window was identified, several formulations parameters were varied to determine their effects on encapsulation yield and residual solvents. This was done using a polymer concentration of 6%. The generated stability window encompassed a wide variety of formulations with different Si oil volumes. These formulations were likely to possess different encapsulation and release characteristics, so the Si oil volume parameter continued to be varied as further characterization progressed.

**Fig. 3:**
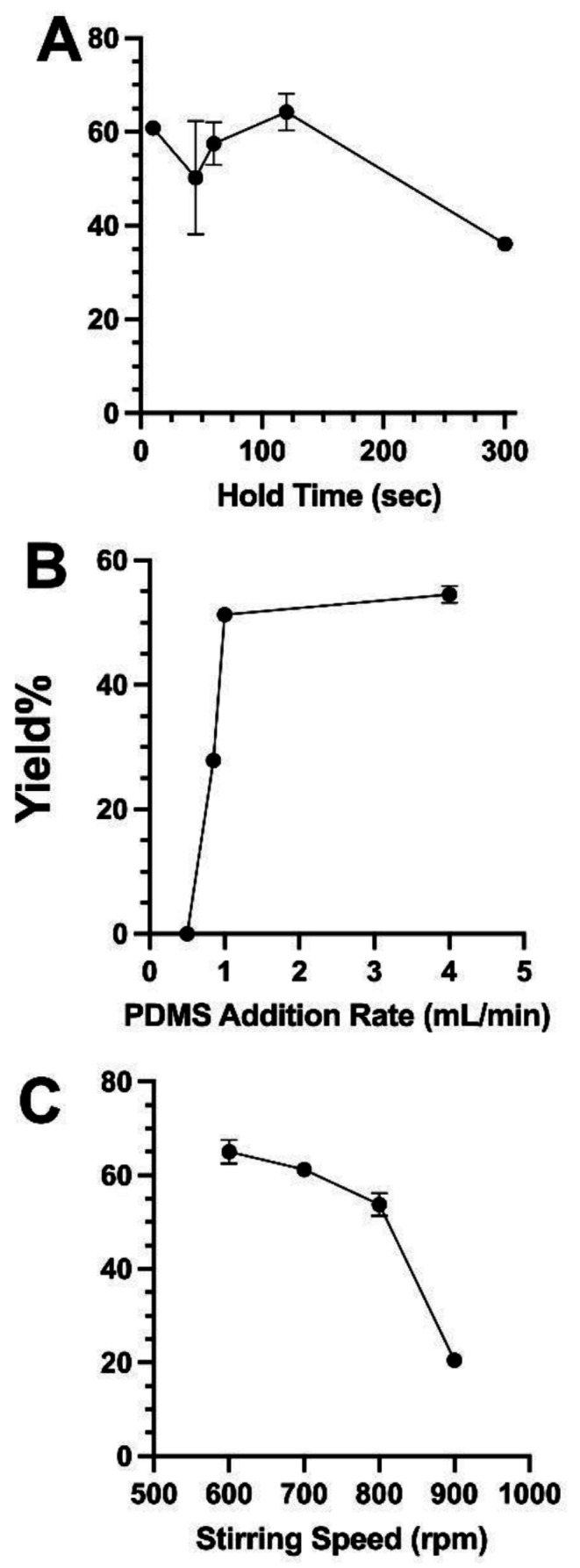
Effect of formulation conditions on encapsulation yield. Varied conditions were (**A**) Hold time, (**B**) PDMS addition rate, and (**C**) heptane bath stirring speed. Values represent *n* = 3 ± SEM. When not varied, conditions held constant were: hold time 60 s, water volume 660 µL, stirring speed 600 rpm, polymer concentration 6% w/v, heptane bath temperature (4 °C), and heptane bath stirring speed (1150 rpm)

The first formulation parameter tested was the length of hold time in between PDMS addition to trigger phase separation, and the heptane wash, to extract the dichloromethane from the newly formed microspheres. As seen in Fig. 3A, there was an extended stability window between roughly 10–120 s where the yield was uniform throughout. By 300 s there was a higher level of polymer aggregation and thus lower yield. At a hold time of 10 min or longer, no functional particles are formed (*data not shown*), with the polymer aggregating into a single mass.

In addition to the total volume of PDMS added to the primary emulsion, the speed at which the PDMS was added was also identified to strongly impact success of properly forming the coacervate. In Fig. 3B, the effect of PDMS addition rate of a total volume of 3.5 mL PDMS on the formulation yield is displayed. Microspheres failed to form at very low addition rates of 0.5 mL/min or lower. This result suggests that the phase separation and initial polymer hardening needs to occur quickly enough otherwise aggregation will occur.

**Fig. 4:**
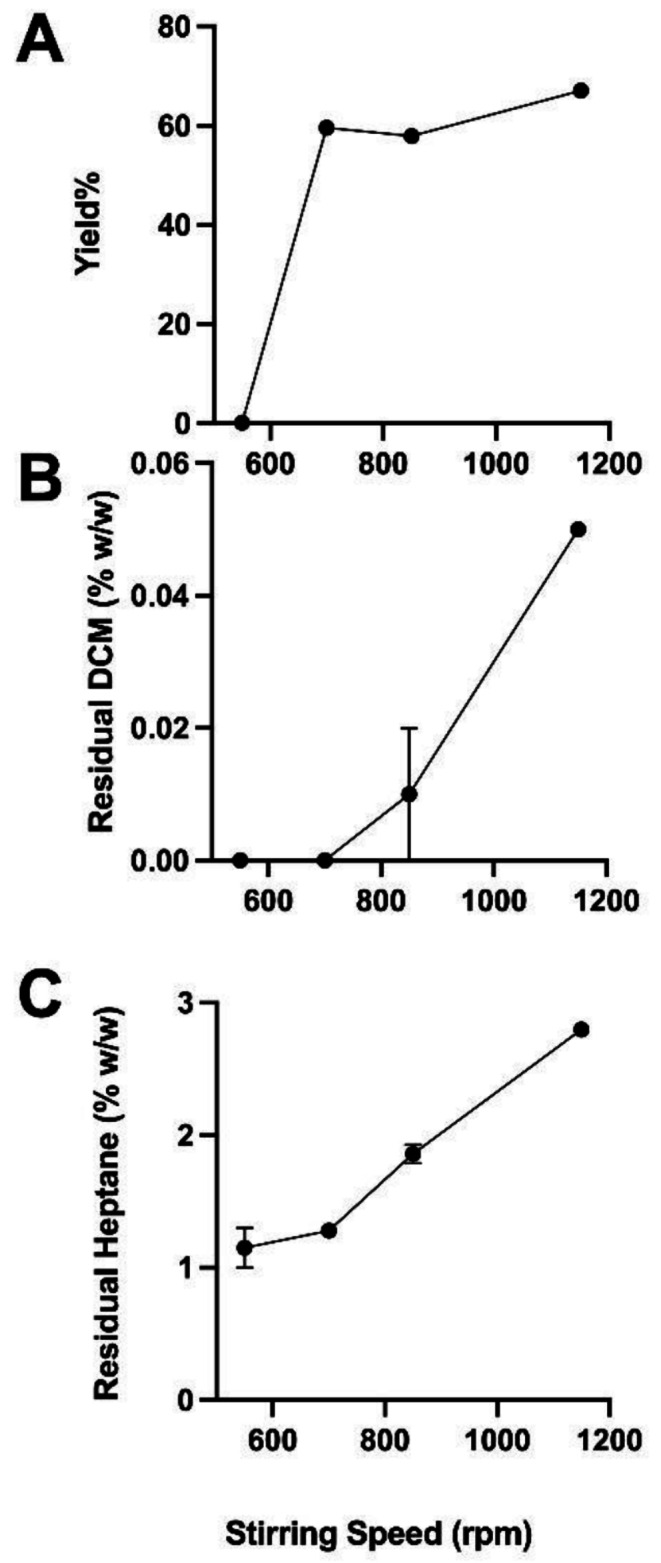
Effect of heptane bath stirring speed on (**A**) yield, (**B**) residual DCM level, and (**C**) residual heptane level. One formulation was made for each stir speed. Residual solvents were measured for *n* = 3 ± SEM. Parameters held constant were polymer concentration (6% w/v), Si oil volume (3.5 mL), water volume (660 µL), hold time (1 min), Si oil addition speed (1 mL/min), stirring speed during Si oil addition (600 rpm), and heptane bath temperature (4 °C)

The third variable examined was the stirring speed during the PDMS dropwise addition step. Figure 3C depicts the encapsulation yield as the stirring speed was increased from 600 rpm to 900 rpm. Stirring speeds between 600 and 800 rpm resulted in reasonably high yields of > 50%. The yield was also influenced by the sieving of particles from 20 to 120 μm. However, when the stirring speed was increased to 900 rpm and beyond, extensive particle aggregation occurred. An explanation for this phenomenon could be related to the formation of deformed polymer matrices. At high spinning speeds, the embryonic microspheres are deformed by the high shear or increased impact frequency with the rotor itself. The high viscosity of the PDMS can aid in stabilizing these deformed microspheres. These deformities increase the contact surface area of the microspheres, which increases the likelihood that another microsphere making contact will adhere to it. Microspheres formulated at high stirring speeds had greatly increased size, as shown in Fig. 6. Additionally, these microspheres were non-spherical due to the high shear in the stirring system.

Other variables such as the inner water: DCM phase volume ratio were examined and found to be of little impact on the yield. The coacervation system described here was capable of accommodating water volumes over the range of 330 to 800 µL with little changes in yield. A possible explanation for this phenomenon is that after sufficient polymer has collapsed around the emulsified water droplets, additional polymer can deposit on the surface without significantly changing the surface characteristics.

In addition to stability window parameters, many key variables govern the washing/drying step after the coacervate is formed. Chiefly among these are the stirring speed and temperature of the heptane bath. The heptane bath is responsible for extracting most of the organic solvents from the polymer and hardening the particles while minimizing particle aggregation.

In Fig. 4A, the effect of stirring speed on microsphere yield is displayed. A single formulation was manufactured for each data point. As seen in the figure, the yield was negligible below 650 rpm. When the stirring speed was between 700 rpm and 1200 rpm the yield reached a maximal value of ~ 60–70%. At rotor speeds above 1200 rpm, immersed air bubbles appeared to have caused aggregation along the new air-heptane interface. As shown in Figs. 4B and C, increasing stirring speed caused increased residual solvent in the particles. This phenomenon was much more apparent for heptane than for DCM. With a 2-fold increase in stirring speed, the residual heptane increased by 2% of the total formulation weight. An explanation for the increased residual solvents at higher stirring speeds is that higher rotor speeds harden the particles quickly. A hardened polymer shell inhibits further solvent removal during the heptane wash and final vacuum drying. This phenomenon has been observed in other methods of formulating microspheres as well [12]. Although formulations that were prepared using a higher stirring speed had elevated residual solvents, these formulations were selected due to higher encapsulation efficiency and lower burst release. Both values of residual heptane and DCM fall within acceptable levels set by the FDA [13].

**Fig. 5:**
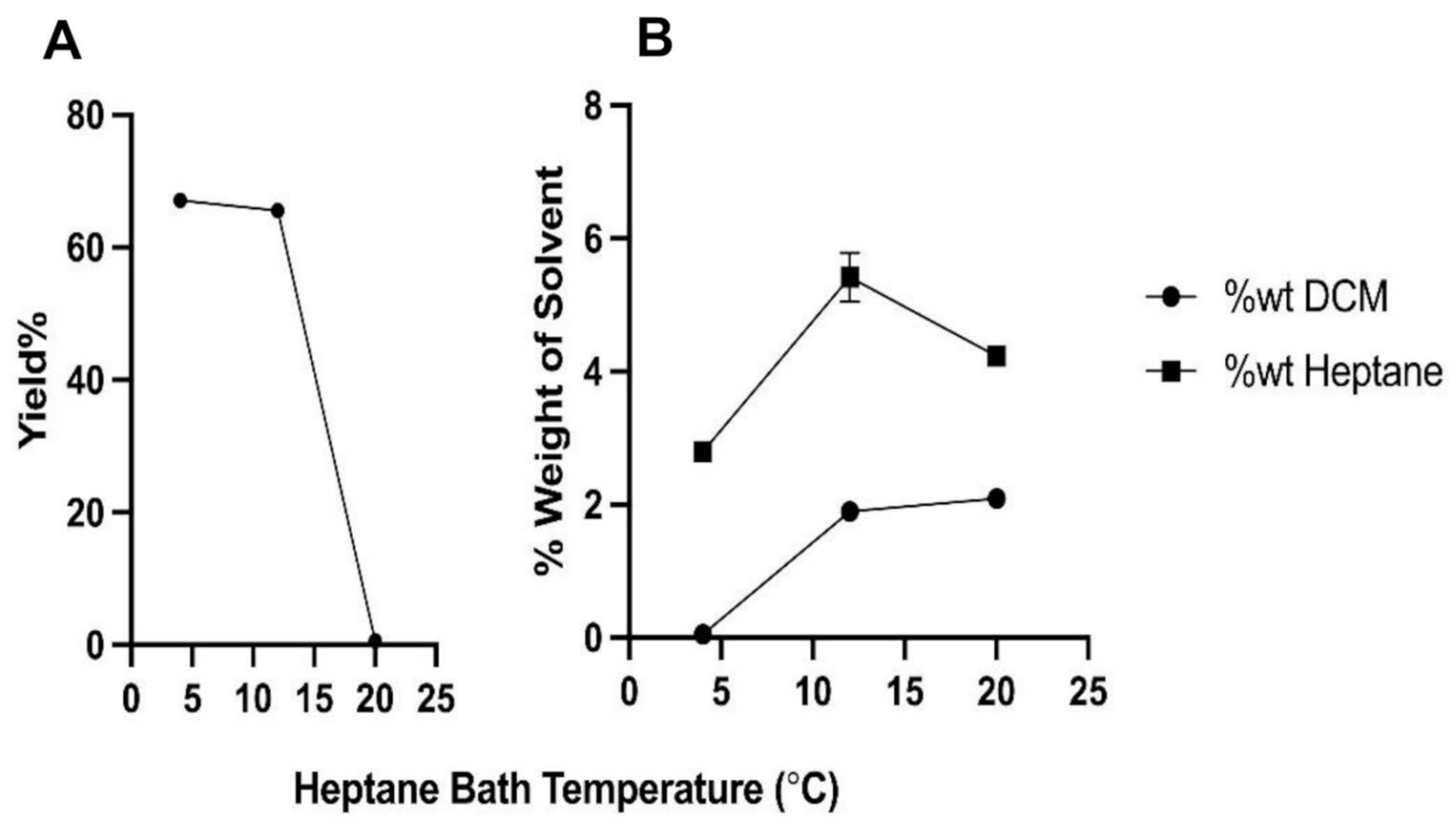
Effect of heptane bath temperature on (**A**) microsphere yield, and (**B**) residual solvents. One formulation was made for each temperature. Residual solvents were measured for *n* = 3 ± SEM. Parameters held constant were polymer concentration (6% w/v), Si oil volume (3.5 mL), and water volume (330 µL), hold time (1 min), Si oil addition speed (1 mL/min), stirring speed during Si oil addition (600 rpm), and heptane bath stirring speed (1150 rpm)

The second variable examined during the heptane wash process was the temperature of the heptane bath (Fig. 5), which was controlled via cooling jacket. When the heptane bath temperature was elevated from 4 to 20 °C, the particles immediately aggregated. Interestingly, the formulation that was washed in a heptane bath at 12 °C avoided aggregation, but still exhibited the elevated residual DCM. The trend that lower heptane temperatures increased the efficiency of DCM removal may seem initially counterintuitive, since the solubility of DCM within heptane is lower at 4 °C compared to that at 20 °C. Since DCM extraction is a diffusion-based process [14], it is likely that the lower temperature slows extraction, and this diminished extraction speed helps avoid formation of an impermeable polymer film on the surface of the polymer microspheres that inhibits further solvent removal.

**Fig. 6:**
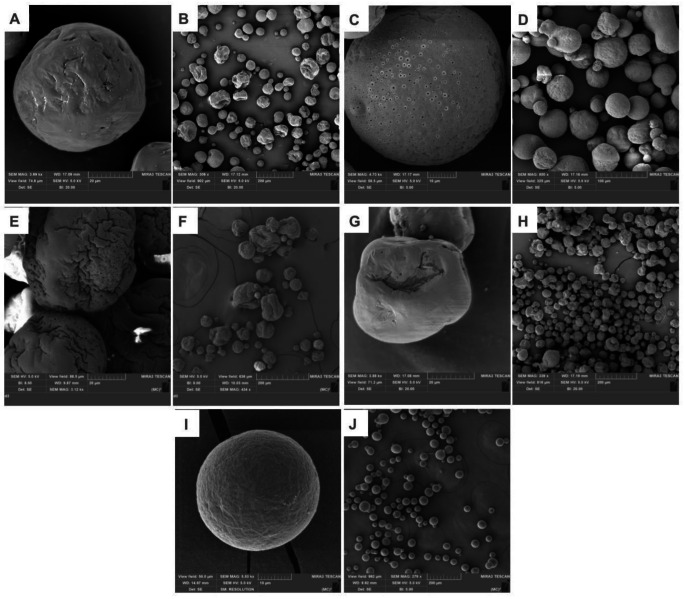
SEM images of various formulations. **6A/6B**: closeup and wide view spectrum of MS with 8% PLGA concentration (55 ± 1.5 µm). **6C/6D**: closeup and wide view spectrum of MS with a hold time of 10 s (55 ± 1.6 µm). **6E/6F**: closeup and wide view spectrum of MS that were stirred at 800 rpm during coacervation (88 ± 3.4 µm). **6G/6H**: closeup and wide view spectrum of MS with a 330 µL water phase (46 ± 0.8 µm). **6I/6J**: closeup and wide view spectrum of MS with optimized parameters: 6% PLGA concentration, hold time of 1 min, stirred at 600 rpm during coacervation, and a 660 µL water phase (56 ± 1.1 µm)

Figure 6I/6J displays SEM images and sizing data for a formulation at 6% PLGA concentration, Si volume of 3.25 mL, water volume of 660 µL, stirring speed of 600 rpm during coacervation and a hold time of 1 min. Each of the other SEM images show one variable deviation from this optimized formulation. For example, the microspheres in Fig. 6A/6B have all the same attributes as the microspheres in 6I/6J except for the PLGA concentration.

The size distribution was consistent across most formulations, with only minimal changes due to batch-to-batch variability. There was a single formulation (6E/6F) that had a much larger size. These microspheres were also deformed and often not spherical, suggesting multiple particle aggregation. Interestingly, the microsphere size distribution includes microspheres slightly lower than 20 μm, despite sieving from 20 to 120 μm. This is likely due to minor microsphere clumping during the heptane wash. Small microspheres can adhere to adjacent microspheres, yet still be small enough to pass through the upper sieve. There is visible microsphere clumping in Fig. 6H, which is likely to occur during the vacuum drying process. The microspheres have a smooth outer matrix, which is interspersed with nanopores at varying densities.

Figure 6A/6B display particles of a higher PLGA concentration than the optimal formulation. The surface morphology displays shallow wrinkles but has a similar appearance to 6I/6J. Figure 6C/6D shows particles with a hold time of 10 s. In the closeup, pores are clearly visible, which accurately represents the higher burst release of these specific particles. Figure 6E/6F show particles that are stirred at 800 rpm during Si oil addition. These particles display visible pores, as well as prominent topographical features. Figure 6G/6F show particles that are formulated with a 330 µL water phase. These particles also have visible wrinkles on the surface, along with several non-spherical deformed particles.

### Effect of formulation variables on encapsulation efficiency and the initial burst release from exenatide-PLGA microspheres

Reduction of burst release from microspheres is commonly an important formulation goal to improve safety and efficacy. In the case of exenatide, an elevated burst release has been known to cause nausea and inflammation at the injection site (2). In addition, a large initial burst reduces the amount of drug available for extended release, thereby reducing the average release rate and/or release period. The encapsulation efficiency (EE), which is often inversely correlated to initial burst, is important for reducing loss of valuable API and ensuring excellent encapsulation. Below the effect of several encapsulation variables on both these parameters was evaluated.

**Fig. 7:**
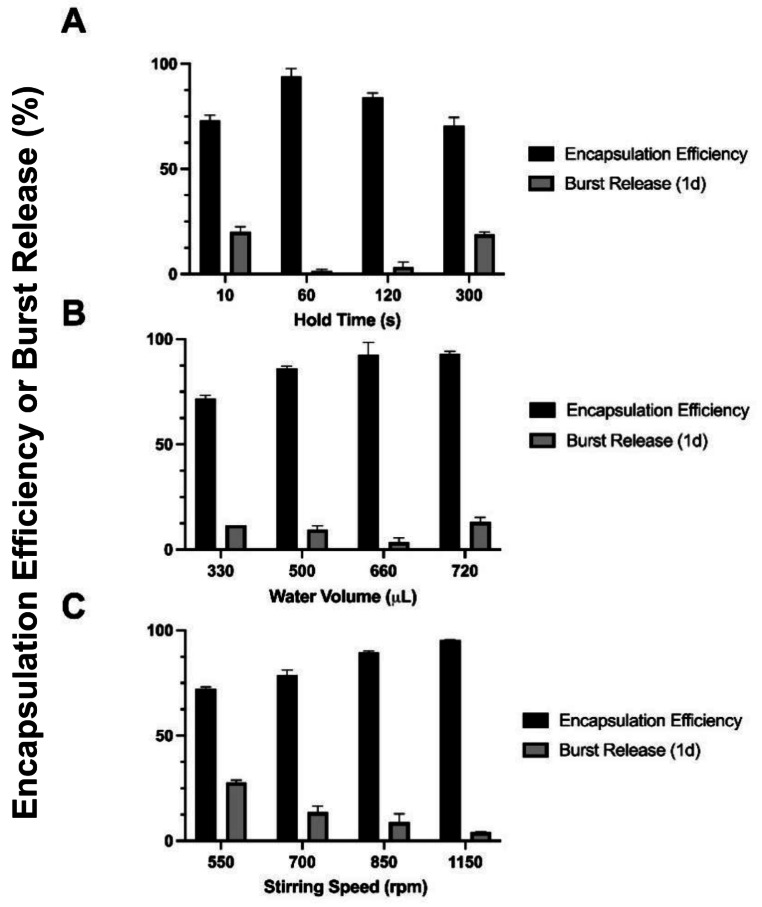
Effect of formulation variables during coacervation encapsulation of exenatide in PLGA microspheres on encapsulation efficiency (EE) and 24-h initial burst release. Formulations variables were (**A**) inner water phase volume, (**B**) hold time, and (**C**) heptane bath stirring speed. Values represent *n* = 3 ± SEM. When not varied variables held constant were: polymer concentration (6% w/v), Si oil volume (3.5 mL), Si oil addition speed (1 mL/min), stirring speed during Si oil addition (600 rpm), heptane bath temperature (4 °C), and heptane bath stirring speed (1150 rpm)

As shown in Fig. 7A, short and long hold times resulted in high burst release and low encapsulation efficiency. A potential reason for this trend could be related to the need for a critical time for the coacervation to be complete but not become unstable. In other words, at low hold times the microparticles have not completely formed and at long times the microparticles began to aggregate. Hold times between 60 and 120 s appeared to be effective at preventing agglomeration during coacervation. At low hold times, it is likely that the phase separation had not been fully completed, thus resulting in incomplete deposition of PLGA onto the water droplets. These resultant microspheres are porous, as seen in Fig. 6C/6D. The higher porosity is likely responsible for the higher burst release. It is unclear why microspheres with a 5 min hold time also exhibited a higher burst release. A hold time of 60–120 s was chosen as the optimal hold time duration because of the low burst release and low aggregation of these formulations.

As seen in Fig. 7B the effect of water phase volume (i.e., the dispersed phase containing exenatide) on EE and the 24-h in vitro burst release under physiological conditions (in PBST at 37 °C) is displayed. As the inner water phase was increased from 330 to 660 µL, the EE rose and burst release dropped. However, increasing the inner water phase slightly above 660 µL to 720 µL did not affect EE but did increase burst release. At lower water volumes, the burst release was comparatively high. Effects of varying the water phase volume are complex. Increasing this volume at constant theoretical drug load caused a decrease in peptide concentration and therefore a reduction in the driving force for diffusion. Higher peptide concentration at low inner water phase volume may also have increased loss of peptide on the homogenization probe. As the water phase volume was increased at a similar primary emulsion size, the number of droplets would be expected to increase, which may have led to emulsion instability. Similarly, increasing the water phase volume is expected to increase size of the emulsion based on the common effect of increasing phase volume ratio causes an increase in emulsion size [15]. Formulations with the highest water volume also displayed a higher burst release. The larger water phase volume may have decreased the diffusion path length during the initial burst phase, thus spurring a quicker release from the pores. Microspheres with a water volume of 330 µL displayed a much smaller particle size, as shown in Fig. 6. However, all the other formulations (490 µL, 660 µL, and 720 µL) displayed similar particle sizes. Therefore, 660 µL was chosen as the optimal water volume value because of the low burst release and high yield of this formulation.

The last variable evaluated for its effect on exenatide encapsulation and burst release was the heptane bath stirring speed. As shown in Fig. 7C, as the stirring speed of the heptane bath was increased, an increase in EE with a simultaneous reduction in initial burst was observed. Since the formulation that was stirred at 1150 rpm possessed the lowest burst release and highest EE, it was selected as the optimal stirring speed.

**Fig. 8:**
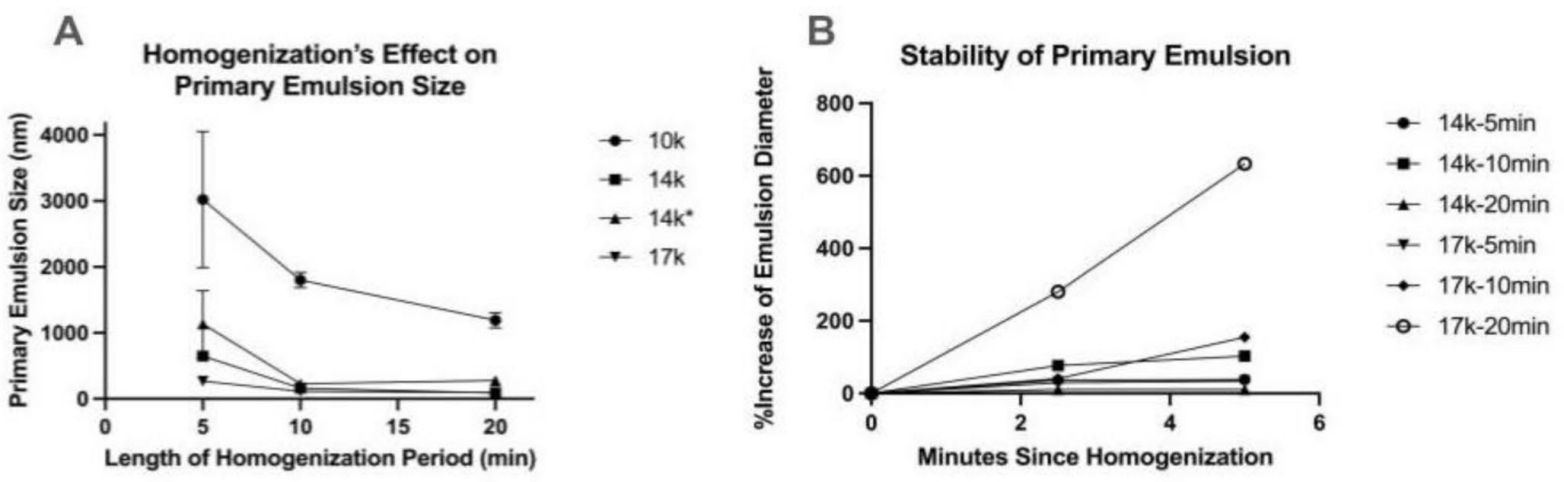
The effects of homogenization on encapsulation and burst release. Effect of homogenization duration and speed on (**A**) primary emulsion size, **B**) kinetics of instability of the primary emulsion after completion of homogenization as a function of homogenization duration and speed). Ten runs were averaged together for each of the data points ± SEM. The mean values from panel A were used for the initial values

As the initial burst of peptides has been linked in part to diffusion through the polymer matrix [16, 17], the primary emulsion size is often a key attribute to controlling burst release [18]. Reducing the primary emulsion size is one way of increasing the average diffusion path length in the polymer matrix and reducing the amount of drug can be released during the initial burst phase. One way to manipulate the primary emulsion size is through changing of homogenization conditions such as speed of mixing and duration of mixing. In Fig. 8A, the effects of three different speeds of homogenization (10k, 14k, and 17k rpm) and three different durations (5, 10, and 20 min) were tested by DLS to measure the primary emulsion size (i.e., volume mean diameter). As expected, all formulations exhibited smaller primary emulsion size as the duration was raised from 5 to 10 min. The subsequent increase from 10 to 20 min did not further decrease the primary emulsion size, except for the 10k formulations. An additional formulation of 14k rpm was performed, to analyze the impact of a hold time between the end of homogenization and analysis. The 14k rpm with a hold time formulation primary emulsion size was larger than that of the 14k rpm without a hold for the 5-min homogenization time but was functionally identical for longer homogenization times.

The stability of these primary emulsions is also important as time is needed to complete the encapsulation. The primary emulsion must be stable for about 5 min while the silicone oil is added to initiate phase separation. The emulsion size was evaluated at 3 and 5 min after homogenization for each of the formulations. Figure 8B displays the temporal stability of the differing formulations as a function of homogenization time and speed. Most of the emulsions showed moderate stability over the short time interval, exhibiting less than an 100% increase in average diameter. The one exception was the formulation with the smallest primary emulsion size, which exhibited a 600% increase in size.

These formulations were evaluated for their initial burst release to ascertain if a relationship between emulsion size and burst release exists, as seen in Table 1. At the end of the 5-min period, the formulations with a primary emulsion diameter of > 1000 nm (10k-5 min, 10k-20 min) exhibited a higher burst release than the primary emulsion diameter of < 1000 nm, and therefore these large primary emulsion formulations were not evaluated further. Any of the 14k-5 min, 14k-20 min, or 17k-5 min formulations were deemed suitable as the final formulation parameter. The 14k-20 min was selected due to the lower standard deviation and better consistency. The success of this formulation suggests that the ideal primary emulsion for controlling burst release should be a sufficiently stabilized over processing time scales, homogenous in its size and have a small diameter. It is also possible that higher homogenization speeds could result in peptide structural damage, although this was not assessed in this study. There was no statistical difference between the exenatide stability during the loading assay. The peptide displayed 98–100% parent peak in the UPLC chromatograms.

**Table 1:**
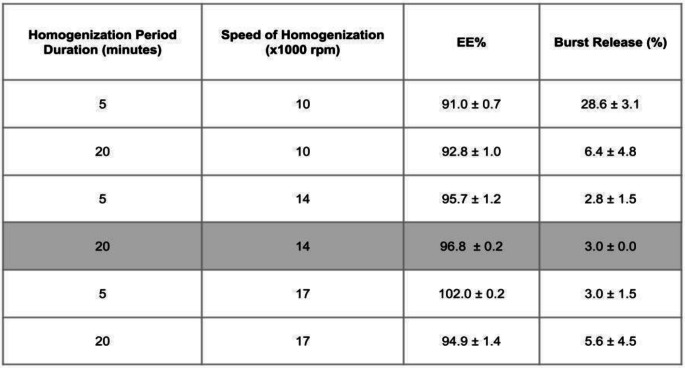
Effect of homogenization duration and speed on exenatide encapsulation efficiency and burst release within 1 day. Values represent *n* = 3 ± SEM

In many double-emulsion formulations made by solvent evaporation, EE is often lower due to exenatide escaping into the outer water layer. For the single emulsion coacervation process, one would expect there to be 100% EE. The drop in EE can be attributed to in most cases to exenatide’s adherence to the homogenization probe. It is worth noting that when a Virtis Cyclone IQ2 homogenizer was used with an identical procedure, the EE was 95–100% compared to 75–85% when the PolyTron homogenizer was used. Therefore, selection of the homogenizer and/or probe would seem to be important to minimize interaction with the peptide.

### Long-term in-vitro release

**Fig. 9:**
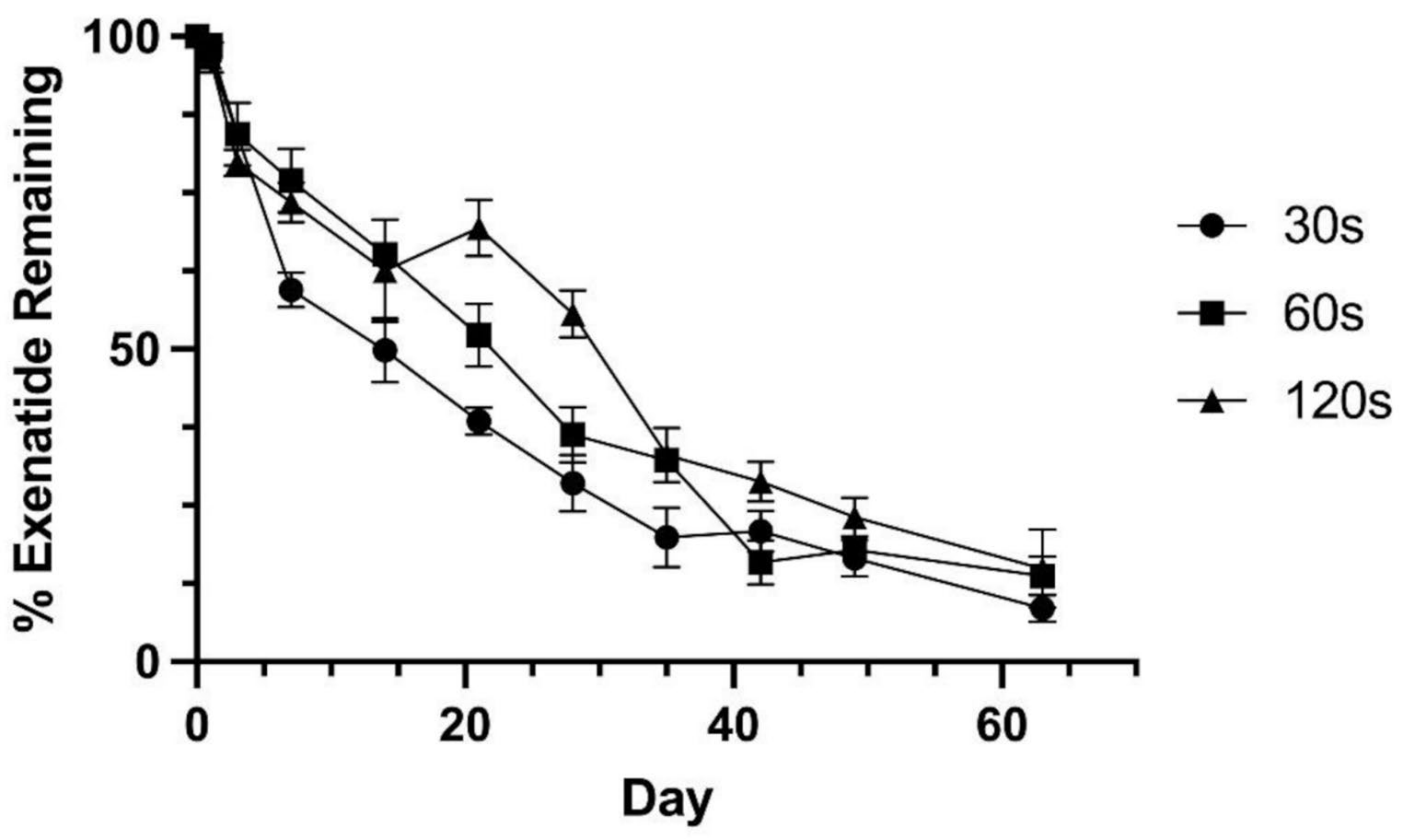
The effect of hold time (30–120 s) during coacervation on the long-term in vitro release kinetics of exenatide from PLGA microspheres. Values represent *n* = 3 ± SEM. Parameters held constant were polymer concentration (6% w/v), Si oil volume (3.5 mL), and water volume (660 µL), Si oil addition speed (1 mL/min), stirring speed during Si oil addition (600 rpm), heptane bath temperature (4 °C), and heptane bath stirring speed (1150 rpm)

Microspheres prepared with various hold times were evaluated for their in vitro release over several weeks to confirm proper encapsulation. As seen in Fig. 9, the formulation with the shortest hold time (30 s) exhibited a faster release profile with ~ 40% exenatide release in the first week. Comparatively, the other formulations with longer hold times displayed only ~ 25% release in the first week. The 30 s hold time formulation maintained the faster release profile until day 42. The 120 s hold time formulation exhibited an interesting feature, in which there appeared to be a brief plateau in release between day 14 and day 21. After day 21, exenatide release was much faster compared to the other formulations until day 42. The 60 s hold time formulation continuously released exenatide at the most constant rate of the three formulations. None of the formulations achieved 100% release of exenatide and stopped releasing peptide after 56 days. This stoppage was likely related to insoluble aggregated peptide [19].

## Conclusions

Coacervation is a commercially used method for encapsulating peptides and small molecules in PLGA microspheres that is sensitive to the formulation conditions. We identified stability windows for maximizing yield empirically by varying process conditions in the absence of expensive APIs. We further defined specific stability windows for our encapsulation apparatus and the specific PLGA and solvent system selected to encapsulate and control the release of the GLP-1 receptor agonist, exenatide, prepared at similar composition to the Bydureon^®^ depot. We identified encapsulation conditions that provided elevated encapsulation of both the peptide and the sucrose stabilizer. Several important relationships were developed between process variables and the encapsulation, microsphere properties (particularly residual organic solvent), and release behavior. Specific formulations were identified that mimic the loading, initial burst, and long-term in vitro release behavior of the commercial formulation. Hold time was found to be one of the most important process parameters, because of its varied effects on yield, burst release, and long-term release. The optimized value of a 60 s hold time provided sufficient time for controlled polymer deposition and formation of homogenous particles without compromising yield. Additionally, a small temporally stable primary emulsion was important to minimize intra-batch and inter-batch variability in EE%. When formulations had a heterogeneous primary emulsion, the resultant particles would exhibit highly variable encapsulation characteristics. Hence, these data may be useful to develop both new and generic peptide-loading microspheres by coacervation.

## Supplementary Information

Below is the link to the electronic supplementary material.

Supplementary Material 1

## Data Availability

All data generated or analyzed during this study are included in this published article and its supplementary information files.
