# Supplementary material for: Formulation and characterization of exenatide-loaded PLGA microspheres prepared by coacervation
Source: Drug Deliv Transl Res. 2025 Dec 9;16(8):2697–710. doi: 10.1007/s13346-025-02008-2 (PMC13346254; doi:10.1007/s13346-025-02008-2)
Supplement: Supplementary file 1 — Supplementary Material 1 [file 13346_2025_2008_MOESM1_ESM.pdf]

Supplementary Information

Formulation and Characterization of Exenatide-loaded PLGA Microspheres Prepared by Coacervation

Cameron White[1] and Steven P. Schwendeman[1,2,*]

[1] Department of Pharmaceutical Sciences and the Biointerfaces Institute

[2] Department of Biomedical Engineering

University of Michigan, Ann Arbor, MI 48109, USA

*Correspondence should be addressed to S. Schwendeman (schwende@umich.edu)

Keywords: Bydureon, PLGA, Coacervation, Phase Separation, GLP-1, Controlled release, initial burst

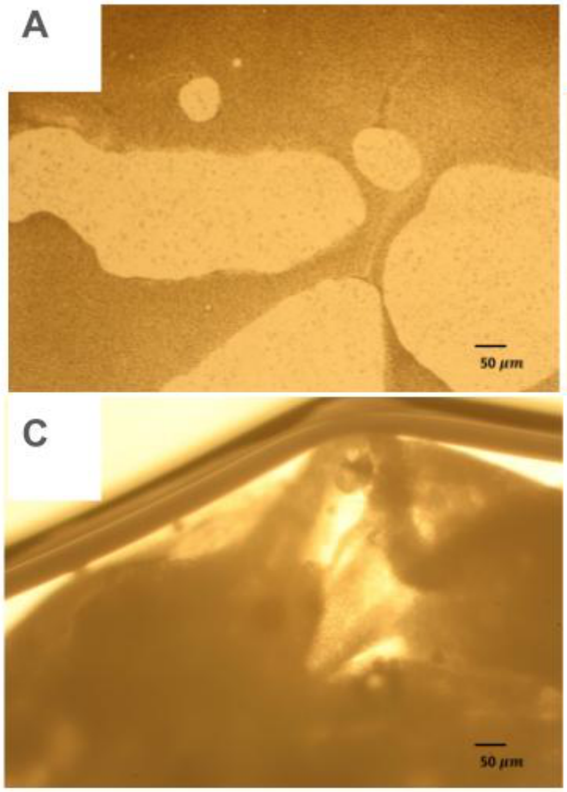

Figure S1: Light microscope images of coacervation formulations before the heptane wash, but after addition of A: 1.25mL Si oil, B: 4mL of Si oil, and C: 15mL of Si oil.

This figure displays the quality of phase separation in Figure S1B, while highlighting the incomplete phase separation in Figure S1A and total polymer precipitation in Figure S1C.

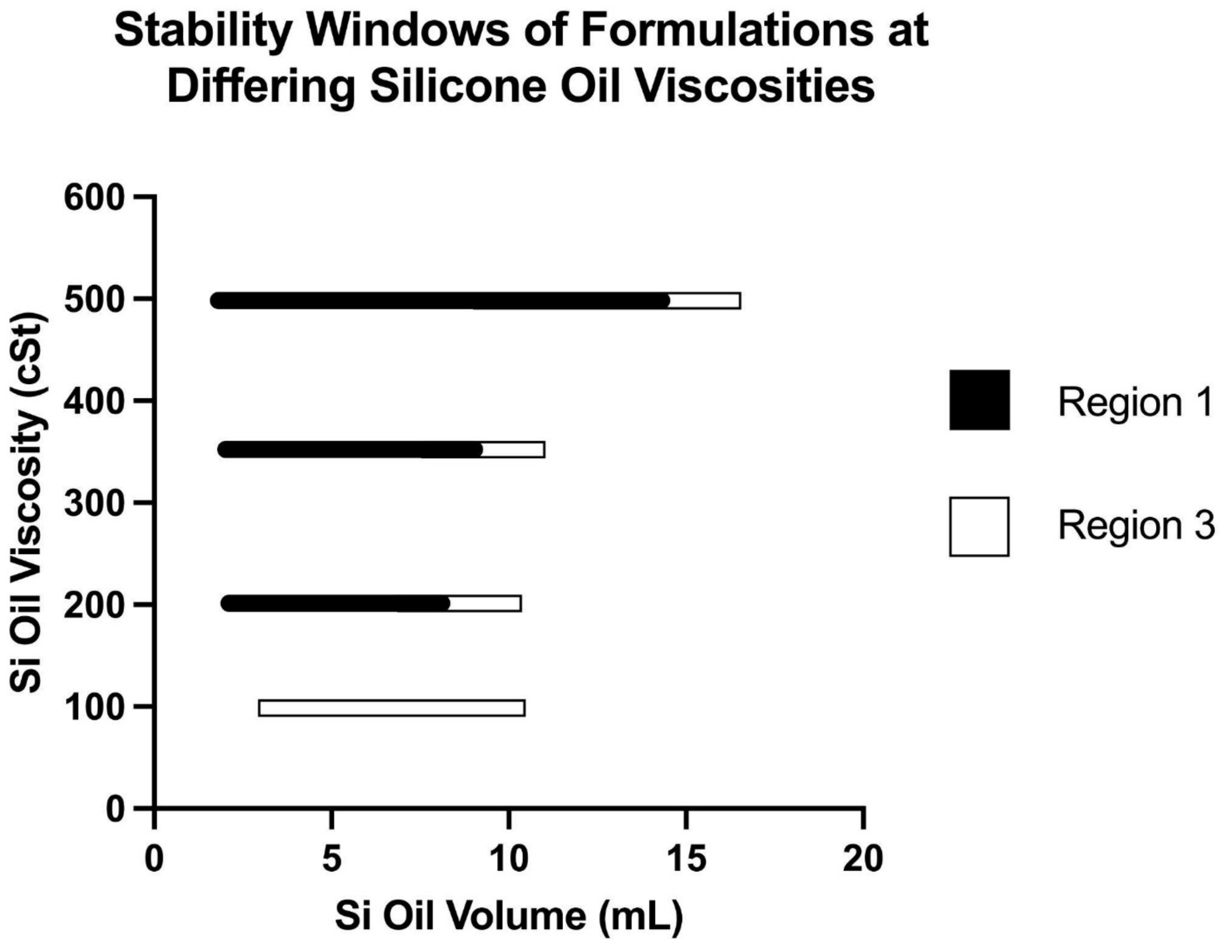

Figure S2: Stability windows of formulations prepared with varying Si oil viscosities.

Region 1: This region represents high yield (>50%) and minimal aggregation or precipitation

Region 3: This region represents lowered yield (25-50%) due to increased aggregation or precipitation

Outside the bars represents volumes of Si oil that failed to produce meaningful quantities of microspheres

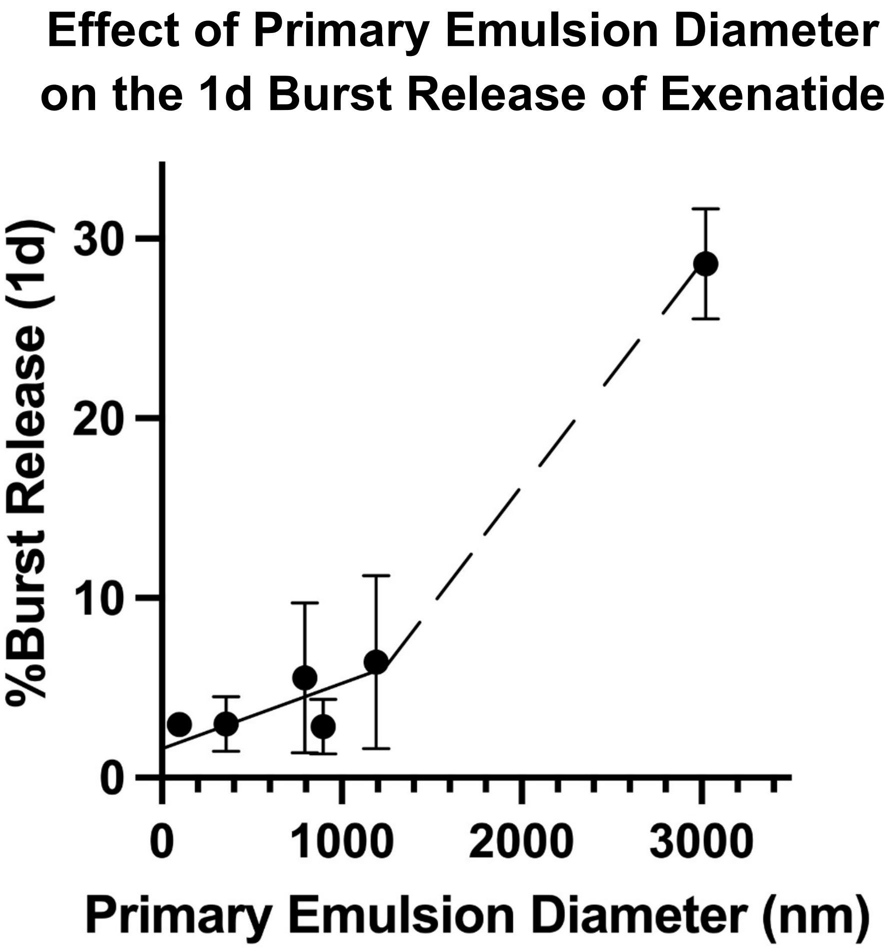

Figure S3: The primary emulsion diameter refers to the size of the primary emulsion 5 minutes after removal from the homogenizer. It is important to note that all the primary emulsions display some coalescence within these 5 minutes. 5 minutes is the typical timeframe for the coacervate to form properly. This figure displays the relationship between the primary emulsion diameter and the burst release of exenatide within the first day. There is a general positive correlation between the two for the first five data points, although several of the data points have high standard deviations. The formulation with the largest primary emulsion diameter has the largest burst release of exenatide (~30%)

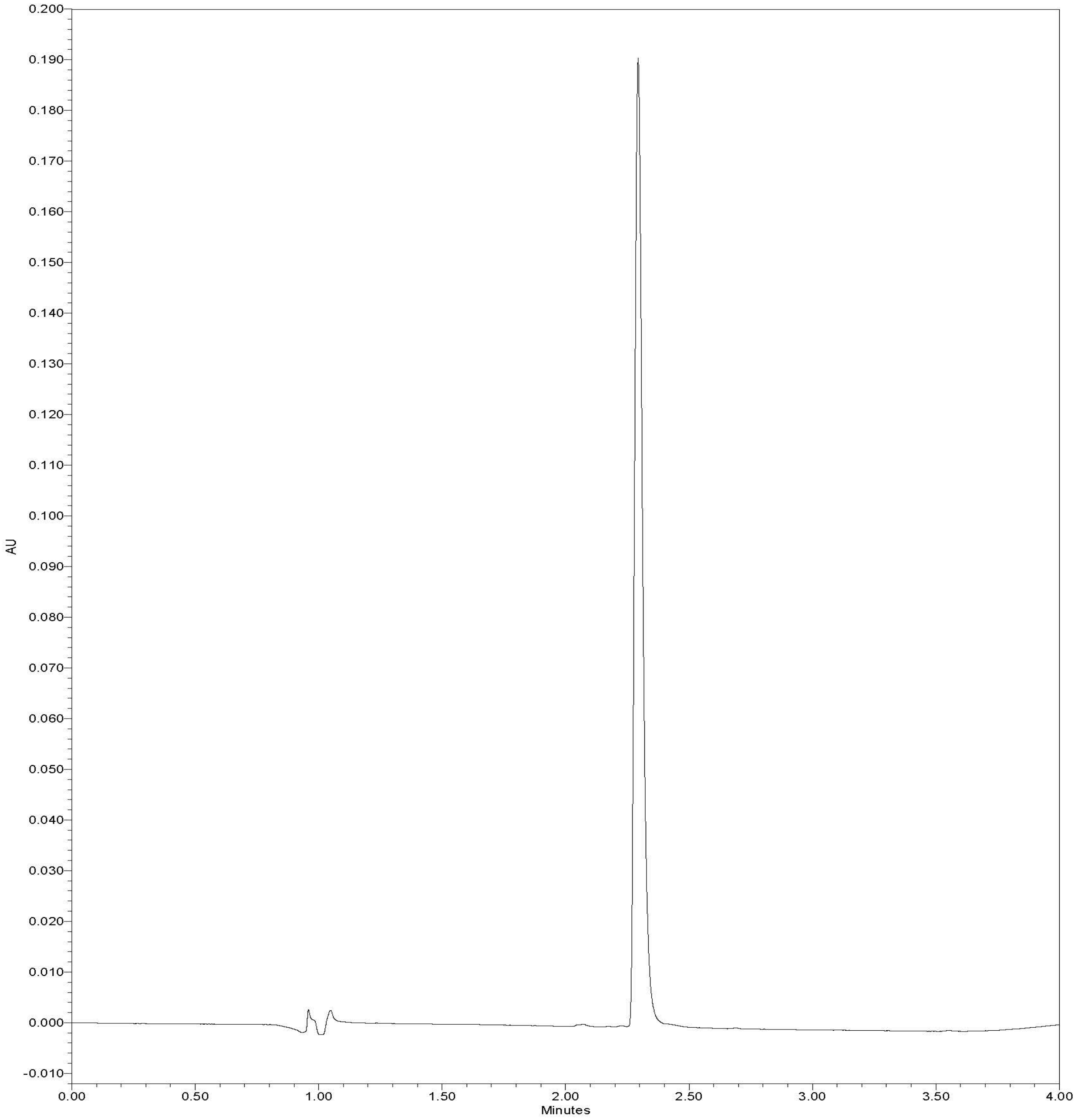

Figure S4: Example UPLC chromatogram of exenatide extracted from PLGA microspheres.

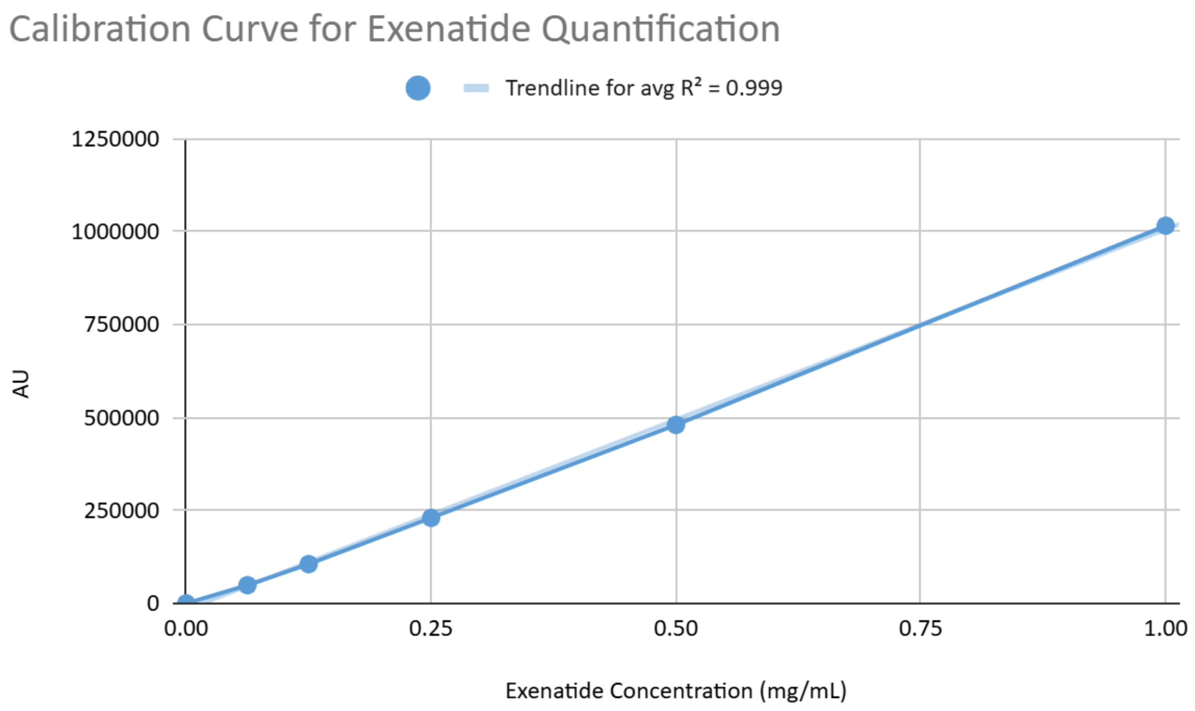

Figure S5: Example UPLC calibration curve for exenatide.
